# Quality and reliability of cardiac rehabilitation-related short Chinese videos on Douyin and Bilibili: a cross-sectional content analysis

**DOI:** 10.3389/fpubh.2026.1797405

**Published:** 2026-06-19

**Authors:** Jie Yuan, Zhuo Chen, Yannan Ma, Song Lin, Xiaodong Feng, Yuzhen Sun

**Affiliations:** 1 Department of Cardiology, Fuwai Central China Cardiovascular Hospital, Zhengzhou, Henan, China; 2 Henan Provincial Hospital of Traditional Chinese Medicine, Zhengzhou, Henan, China; 3 Department of Rehabilitation Medicine, The First Affiliated Hospital of Henan University of Chinese Medicine, Zhengzhou, Henan, China

**Keywords:** cardiac rehabilitation, exercise rehabilitation, public education, public health, public media

## Abstract

**Background:**

Social media platforms have emerged as prominent channels for disseminating cardiovascular health information. However, the accuracy, completeness, and clinical reliability of cardiac rehabilitation (CR)-related content vary widely. Therefore, this study This study aims to identify upload sources, contents, and feature information of these videos on Bilibili and Douyin, and further evaluate the video quality.

**Methods:**

A cross-sectional study was conducted on Bilibili and Douyin using the keywords “cardiac rehabilitation (心脏康复)” and “postoperative coronary heart disease (冠心病术后康复).” A total of 200 videos were included. Data on video characteristics were collected, including title, uploader identity, upload time, video duration, content type, engagement metrics (likes, comments, and shares), presentation format, and video quality scores. Video quality and reliability were assessed using the modified DISCERN (mDISCERN), the Journal of the American Medical Association (JAMA) benchmark criteria, and the Global Quality Scale (GQS).

**Results:**

Douyin videos were shorter in duration and more recently uploaded, and received significantly more likes and comments than Bilibili videos (*P* < 0.001). No significant differences were observed between platforms in terms of saves or shares. Engagement metrics differed significantly across uploader categories for likes and comments (*P* < 0.001). Videos uploaded by hospital departments achieved the highest mDISCERN, JAMA, and GQS scores (*P* < 0.01). Bilibili videos showed slightly higher JAMA scores than Douyin videos (*P* = 0.04). Spearman correlation analysis indicated that content quantity was positively associated with both GQS and JAMA scores. Overall, associations between video quality scores and user engagement metrics were weak and inconsistent. GQS showed a weak positive correlation with shares, whereas mDISCERN was negatively correlated with comment counts. No significant association was observed between JAMA scores and engagement indicators.

**Conclusions:**

Our study shows that the quality of short videos on health information related to CR is poor on Bilibili and Douyin. However, videos uploaded by institutional and healthcare professional accounts demonstrate better performance in terms of information reliability and content quality. Therefore, CR information obtained from short-video platforms should be interpreted with caution, and viewers are advised to critically evaluate content credibility before using such information to guide health-related decisions.

## Introduction

Cardiac rehabilitation (CR) has been shown to effectively improve cardiac function in patients with cardiovascular disease while significantly reducing the risk of disease recurrence and mortality. Nevertheless, public and patient awareness of CR, as well as actual participation rates, remain persistently low. In a survey of 500 patients with coronary heart disease in China, only 47.3% reported being aware of CR (1). Studies from different countries and regions have consistently identified limited knowledge of CR as a common barrier to patient participation worldwide (2). Accordingly, enhancing public awareness and understanding of CR is crucial for supporting long-term disease management and effective secondary prevention among patients with coronary heart disease (3, 4). Video has become a widely used medium for health communication, as it integrates visual and verbal information and facilitates the presentation of complex concepts in a more accessible and memorable manner (5–7). With the rapid proliferation of the internet, video-sharing platforms have emerged as a primary source of health information not only for the general public, but also for healthcare professionals and students alike (8). However, popularity does not necessarily reflect quality. Videos with high viewership may still contain incomplete, biased, or unverified information, underscoring the urgent need for systematic evaluation and oversight of online health content (9). In China, Douyin and Bilibili represent two of the most prominent platforms for short-form health video content. While Bilibili initially gained popularity among younger audiences, it has since expanded to host a diverse range of health-related content, encompassing chronic disease management, rehabilitation guidance, and patient education (10). Douyin, launched in 2016, has rapidly expanded its user base and supports the wide dissemination of short, highly engaging health videos (11). Previous research has assessed the quality of health-related videos across a range of conditions, including liver cancer (10), esophageal cancer (12), gastroesophageal reflux disease (13), chronic obstructive pulmonary disease (14), diabetes (15), and several surgical topics such as gallstones (16) and lung nodules (17). These studies consistently report considerable variation in information quality and reliability. Content produced by healthcare professionals or institutions tends to score higher, whereas videos from non-professional sources—despite often attracting substantial engagement—are more likely to contain incomplete or misleading information (18).

Research on video content related to CR remains scarce and has predominantly focused on a single platform, most commonly YouTube. Previous studies (19, 20) have shown that the overall quality of such videos is generally low to moderate when evaluated using standardized assessment tools, including the modified DISCERN (mDISCERN), the Journal of the American Medical Association (JAMA) benchmark criteria, and the Global Quality Scale (GQS). In addition, video quality varies by source, with content produced by healthcare professionals or institutional accounts typically receiving higher quality scores. Nevertheless, several limitations persist within the existing literature. Most studies have been based on single-platform data, which limits the ability to reflect the diversity of the contemporary short-video ecosystem and hinders cross-platform comparisons of content characteristics, presentation formats, and information quality. Furthermore, prior research has focused predominantly on informational quality, with relatively little attention paid to user engagement and its association with objective quality indicators. Similarly, variations in engagement levels across different uploader types remain underexplored. Against the backdrop of the growing importance of short-video platforms such as Douyin and Bilibili as key sources of health information, these research gaps have become increasingly evident. Accordingly, the present study adopted a cross-platform design to investigate CR–related videos on Douyin and Bilibili. Video quality was assessed using the mDISCERN, the JAMA benchmark criteria, and the GQS. The associations between video quality and uploader type, video duration, and user engagement metrics—including likes, comments, shares, and favorites—were further examined. This study aims to provide a comprehensive characterization of the quality and dissemination patterns of CR–related video content on major Chinese short-video platforms.

## Methods

### Search and data collection

**Figure 1:**
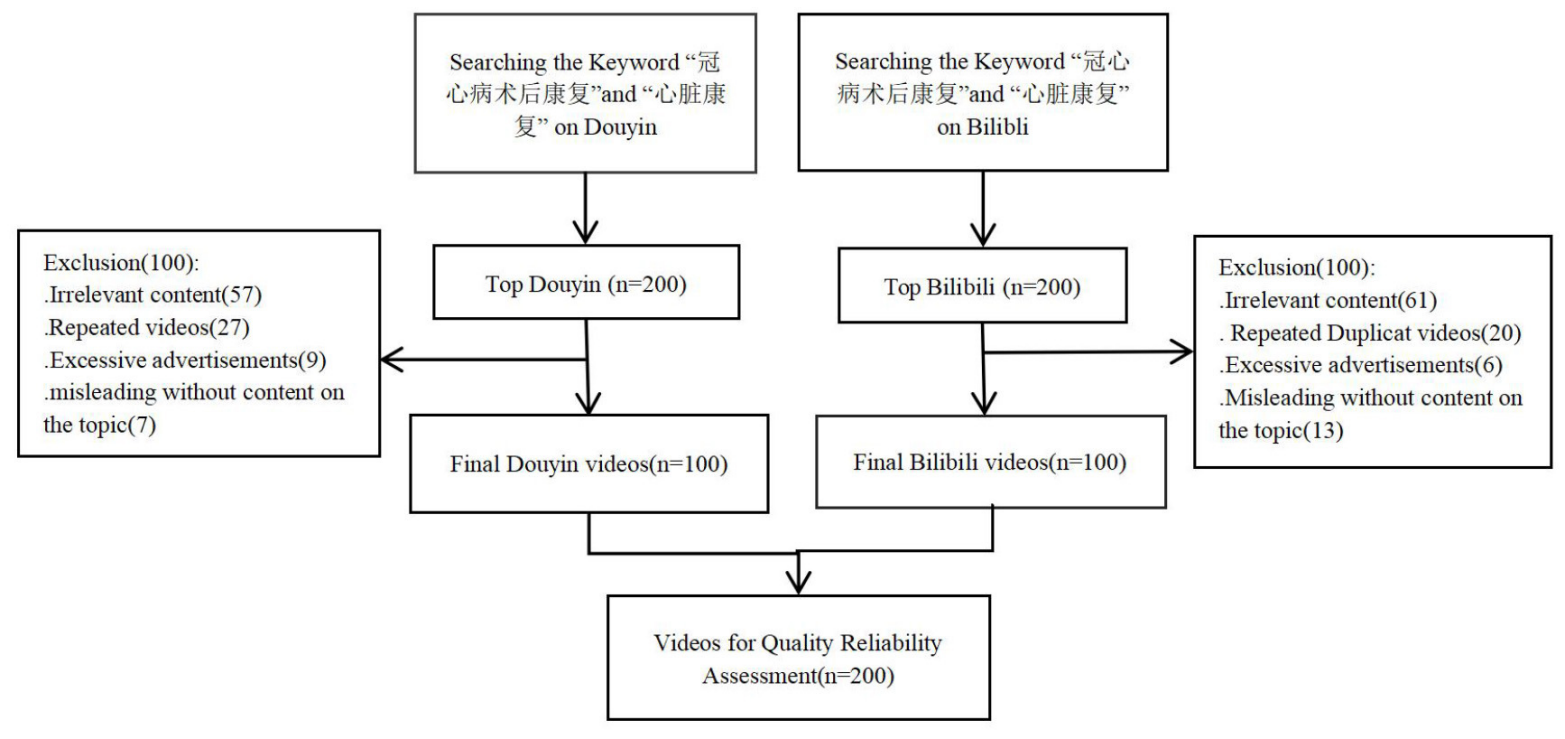
Search strategy for short videos on cardiac rehabilitation.

On 29 October 2025, video retrieval was conducted separately on Bilibili and Douyin using two pre-defined Chinese keywords: “冠心病术后康复” (post-coronary heart disease surgery rehabilitation) and “心脏康复” (cardiac rehabilitation). Each keyword was searched independently on both platforms. Before searching, all accounts were logged out and search history was cleared to minimize personalization bias. No additional filters were applied, and all searches were performed using the default ranking order provided by each platform. For each platform, the top 100 videos returned for each keyword were retrieved, resulting in 200 videos per platform for initial screening. Duplicate videos arising from overlap between the two keyword searches were identified by matching video URLs and titles and were removed prior to screening. On Douyin, 27 duplicate records were removed. Subsequently, 57 videos unrelated to CR, seven videos containing only static images or text without audiovisual content, and nine videos with excessive promotional content were excluded. The final sample included 100 Douyin videos. A similar procedure was applied to Bilibili. After duplicate removal (*n* = 20), 61 unrelated videos, six promotional videos, and 13 videos not specifically focused on CR or considered potentially misleading were excluded. The final sample included 100 Bilibili videos. The screening process is summarized in Figure 1. For all included videos, data were extracted on title, uploader identity, video duration, content type, engagement metrics (likes, comments, and shares), presentation format, and video quality scores. Video quality and reliability were assessed using the mDISCERN, the JAMA, and the GQS. All data were organized in Microsoft Excel for subsequent analysis.

### Video parameters

For each video, a comprehensive dataset was collected, including uploader identity, upload date, video duration, view count, content category, quality score, presentation style, and engagement metrics (likes, comments, favorites, and shares). To adjust for disparities in posting duration, engagement metrics were normalized based on the number of days between video upload and data collection, and further standardized to a 30-day period to yield comparable interaction rates (i.e., likes, comments, and shares per 30 days). These variables were used in subsequent between-group comparisons. Notably, total view count data were not available for Douyin videos. Based on uploader identity, videos were classified into five categories: Physicians, comprising professionally certified individual accounts; Other healthcare professionals, including rehabilitation therapists, nurses, and allied health personnel; Hospital departments, representing officially certified institutional accounts; Individual users, encompassing uncertified accounts such as reposted content without clear attribution and patient-shared videos; and Official media, consisting of officially certified media outlets.

Based on videos content, videos were classified into eight categories: Basic rehabilitation knowledge: definition of CR, goals of CR, phases of CR, and key precautions; Exercise prescription: types of exercise, exercise frequency, and intensity monitoring; Diet and nutrition; Smoking cessation and alcohol reduction; Complication prevention and health management: including post-procedural restenosis, arrhythmias, and control of blood pressure and blood lipids; Risk factor control and monitoring: introduction of methods for monitoring blood pressure, blood glucose, body weight, and heart rate; Medication management: including education on antiplatelet agents, β-blockers, statins, and other medications; Rehabilitation follow-up: home-based and community-based rehabilitation, and follow-up arrangements. This classification is highly consistent with the core components of CR and secondary prevention as outlined in international guidelines issued by the AHA, ESC, and WHO (21, 22). A multi-label coding approach was employed in the present study. Videos addressing multiple topics were assigned to more than one thematic category simultaneously. For each platform, the proportion of each topic category was calculated as the number of videos assigned to that category divided by the total number of topic assignments across all categories on that platform. As multiple labels could be assigned to a single video, the total number of category assignments exceeded the number of included videos.

### Ethical considerations

All data analyzed in this study were obtained from publicly accessible video-sharing platforms (Douyin and Bilibili). The study did not involve any direct interaction with individuals, nor did it include the collection of personal or identifiable information, or any form of intervention. Therefore, in accordance with institutional policies and applicable regulations, this study was exempt from ethical review and ethical approval from an institutional review board was not required.

### Quality and reliability assessment instruments

The mDISCERN instrument was used to assess the reliability and structural quality of the information presented in the health education videos. This tool was adapted from the original mDISCERN questionnaire developed by Charnock et al. (23) and refined by Singh et al. (24). Each item was scored dichotomously (1 = “Yes,” 0 = “No”), resulting in a total score ranging from 0 to 5, with higher scores indicating greater informational reliability. The instrument evaluates whether: (1) the aims of the video are clearly stated and achieved; (2) the information is supported by reliable, evidence-based sources; (3) the content is presented in a balanced and unbiased manner; (4) additional references or sources of information are provided; and (5) areas of uncertainty or limitations are acknowledged.

The Journal of the JAMA criteria are used to evaluate the transparency, traceability, and academic accountability of online health information. First proposed by Silberg et al. (25), this framework has since been widely applied in assessing the quality of medical websites and video content. It consists of four dimensions: authorship, attribution, disclosure, and currency. Each criterion meeting the standard is awarded 1 point, yielding a total possible score ranging from 0 to 4. A higher score indicates greater transparency and credibility of the information source.

The GQS is used to comprehensively evaluate the overall educational quality, information fluency, and patient utility of videos. Initially proposed by Bernard et al. (26). The GQS rating criteria are as follows: Level 1 indicates poor quality, with very little information and limited help for patients; Level 2 represents moderate quality with some useful content but overall suboptimal delivery; Level 3 represents also medium quality, with sufficient presentation of key information; Level 4 denotes high quality, with comprehensive content and strong practicality; Level 5 indicates excellent quality, with extremely high information value for patients. Scores ranged from 1 to 5, where 1 indicated “poor quality with little to no practical value” and 5 indicated “excellent quality that is comprehensive and highly practical.

### Evaluation process

All included videos were independently evaluated by two senior CR specialists (SL and DXF), each with more than 10 years of clinical experience in cardiovascular rehabilitation. Prior to evaluation, both reviewers completed standardized training on the mDISCERN, JAMA benchmark criteria, and GQS instruments. Inter-rater reliability was quantified using the intraclass correlation coefficient (ICC) based on a two-way mixed-effects model. In cases of disagreement, discrepancies were first resolved through discussion and consensus; if consensus could not be reached, a third reviewer (ZC) made the final decision. A standardized coding framework was developed based on pre-defined operational definitions, encompassing all categorical variables including uploader classification, content themes, and presentation style. Uploader type and presentation format were classified with reference to platform context, video characteristics, and methodologies adopted in prior video-based health information research. Content themes, by contrast, were defined in strict accordance with the core components of CR and secondary prevention as outlined in international guidelines issued by the American Heart Association (AHA), the European Society of Cardiology (ESC), and the World Health Organization (WHO). Prior to formal data extraction, both reviewers jointly conducted a pilot assessment of 40 pre-screened videos, after which the coding framework was iteratively refined through discussion and consensus to enhance the clarity, consistency, and applicability of all category definitions. The finalized framework was subsequently applied to the full set of included videos.

### Statistical analysis

All statistical analyses were performed using R software (version 4.5.0; R Foundation for Statistical Computing, Vienna, Austria) (41). The Shapiro–Wilk test was used to assess the normality of continuous variables. Normally distributed variables are presented as mean ± standard deviation (SD), whereas non-normally distributed variables are expressed as median (interquartile range, IQR). Group comparisons between Douyin and Bilibili were performed using the Wilcoxon rank-sum test. The Kruskal–Wallis test was used to assess overall differences in user engagement metrics across uploader categories. When a significant overall effect was observed, *post-hoc* pairwise comparisons were performed using Wilcoxon rank-sum tests with Bonferroni correction. Categorical variables (including uploader types, presentation styles, and content topic categories) are presented as frequencies and percentages, and were compared using the chi-square test or Fisher's exact test, as appropriate. Inter-rater reliability was assessed using the intraclass correlation coefficient (ICC). ICC values were interpreted as follows: values <0.50 indicated poor reliability, 0.50–0.75 indicated moderate reliability, 0.75–0.90 indicated good reliability, and >0.90 indicated excellent reliability. Spearman correlation analysis was conducted to evaluate the associations between audience engagement metrics (such as likes, comments, and saves) and video number and reliability scores (mDISCERN, JAMA, and GQS). A two-tailed *P*-value <0.05 was considered statistically significant.

## Result

### Video screening and characteristics

Figure 1 illustrates the process of video retrieval and selection. A total of 400 videos were initially screened, and after rigorous assessment and application of pre-defined exclusion criteria, 200 videos were included for analysis, comprising 100 from Douyin and 100 from Bilibili. As detailed in Table 1, Douyin videos were shorter in duration than Bilibili videos (*P* < 0.001) and were uploaded more recently (*P* < 0.001). In terms of engagement metrics, Douyin videos had significantly higher median likes (150.00 vs. 23.00, *P* < 0.001) and comments (8.50 vs. 1.00, *P* < 0.001), whereas no significant difference was observed in the number of saves between the two platforms (32.00 vs. 65.00, *P* = 0.33). Bilibili videos had higher median shares than Douyin videos (54.50 vs. 31.00, *P* = 0.512), although this difference was not statistically significant. After normalization by upload time, Douyin videos demonstrated significantly higher engagement rates across all indicators, including likes, comments, saves, and shares per 30 days (*P* < 0.001). Overall, compared with Bilibili, Douyin videos tended to be shorter, more recently uploaded, and generated substantially higher time-standardized user engagement.

**Table 1:** Characteristics of videos on Bilibili and Douyin.

Video parameter	Overall (*n* = 200)	Douyin (*n* = 100)	Bilibili (*n* = 100)	*P*
Length, min	2.57 (1.28–8.48)	1.35 (1.00–2.10)	7.25 (3.00–26.38)	<0.001
Upload time, day	677.00 (207.00–1303.00)	218.50 (103.25–475.25)	1268.50 (924.00–1620.25)	<0.001
Likes, *n*	42.50 (18.00–202.25)	150.50 (34.25–763.50)	23.00 (7.25–47.00)	<0.001
Total views	–	–	2139.50 (727.75–4077.00)	–
Likes/30 days	2.37 (0.56–21.63)	19.60 (4.54–168.69)	0.59 (0.18–1.12)	<0.001
Saves, *n*	60.00 (11.00–207.75)	32.00 (8.25–220)	65.00 (26.25–205.25)	0.33
Saves/30 days	2.73 (0.85–9.09)	4.57 (1.25–32.02)	1.64 (0.62–4.22)	<0.001
Comments, *n*	2.50 (1.00–15.75)	8.50 (2.00–39.25)	1.00 (0.00–3.00)	<0.001
Comments/30 days	1.03 (0.17–1.39)	6.15 (1.11–75.78)	0.22 (0.00–0.08)	<0.001
Shares, *n*	44.00 (7.25–141.00)	31.00 (4.25–235.00)	54.50 (12.25–106.00)	0.512
Shares/30 days	1.68 (0.54–5.12)	3.40 (0.80–23.89)	1.18 (0.31–3.05)	<0.001

Values are presented as median (interquartile range, IQR). Group comparisons between Douyin and Bilibili were performed using the Wilcoxon rank-sum test. A two-tailed *P*-value <0.05 was considered statistically significant.

### Uploader characteristics

**Figure 2:**
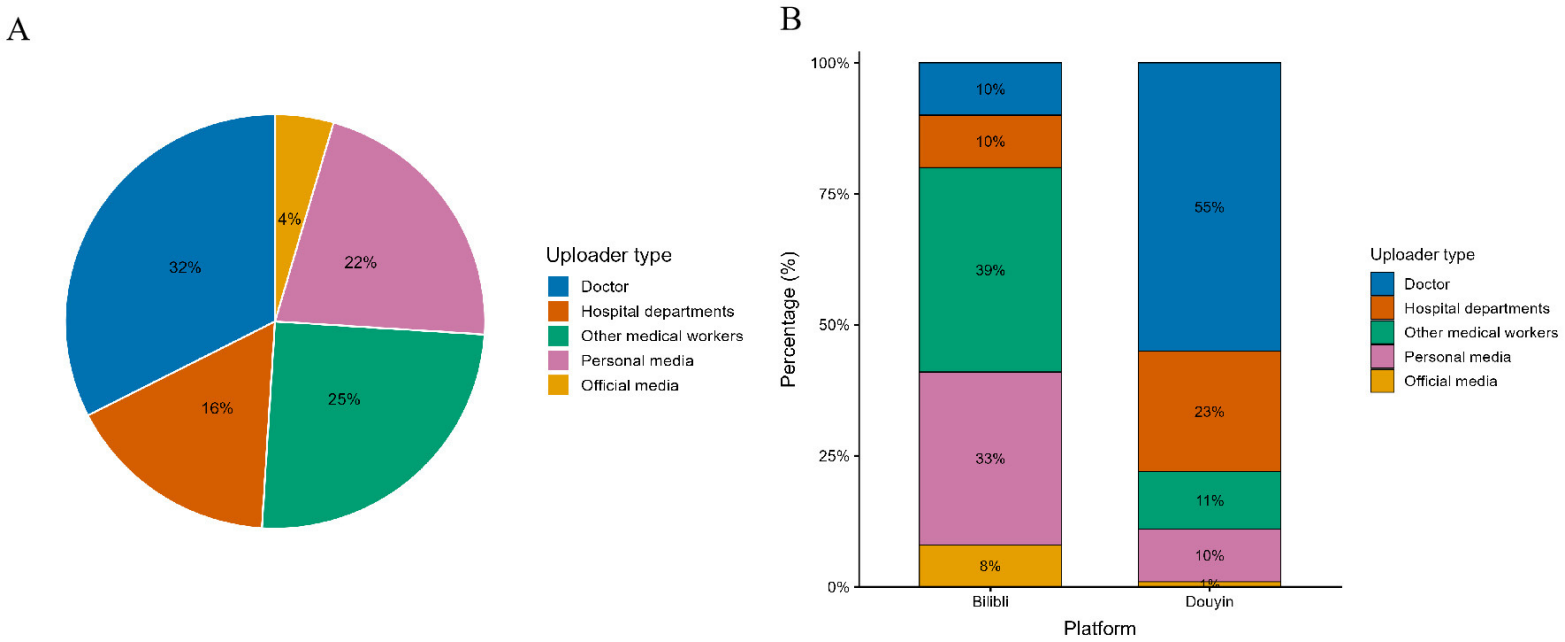
Proportion of different uploader types. **(A)** Percentage of all uploader types. **(B)** Comparison of uploader type percentages between Douyin and Bilibili.

As shown in Figure 2, the uploader types on Douyin and Bilibili, the doctor represented the predominant group of uploaders (*n* = 65), followed by personal media accounts (*n* = 43), with official media contributing the fewest videos (*n* = 9). On Douyin, doctor constituted the majority of uploads (55%), followed by hospital departments (23%). In contrast, on Bilibili, other medical workers uploaded the largest share of videos (39%), with personal media and individual sharing accounting for 33%. Table 2 shows that video engagement metrics vary by uploader category. Videos uploaded by doctors showed the highest median number of likes 187.00 (IQR: 27.00–754.50) and comments 8.00 (IQR: 2.00–41.00). Significant overall differences among uploader categories were observed for likes and comments (*P* < 0.05), whereas no significant differences were found for saves or shares (*P* > 0.05). *Post-hoc* pairwise comparisons with Bonferroni correction further revealed that doctor-uploaded videos had significantly higher likes and comments compared with other medical workers and personal media accounts (*P* < 0.05).

**Table 2:** Characteristics of uploader types of videos on Douyin and Bilibili.

Video metrics	Total (*n* = 200)	Doctor (*n* = 65)	Other medical worker (*n* = 50)	Personal media (*n* = 43)	Hosipital department (*n* = 33)	Official media (*n* = 9)	*P-*value
Likes, *n*	42.50 (18.00–202.25)	187.00 (27.00–754.50)	30.00 (11.50–52.50)	31.00 (9.00–148)	52.00 (19.00–158.00)	23.00 (1.50–190.50)	<0.001
Saves, *n*	60.00 (11.00–207.75)	27.00 (6.00–305.00)	68.00 (12.50–201.75)	65.00 (23.00–237.50)	52.00 (15.00–149.50)	62.00 (2.50–554.50)	0.588
Comments, *n*	2.50 (1.00–15.75)	8.00 (2.00–41.00)	1.00 (0.75–3.0)	1.00 (0.00–10.50)	2.00 (1.00–10.00)	1.00 (0.50–24.00)	<0.001
Shares, *n*	44.00 (7.25–141.00)	13.00 (4.50–275.00)	31.50 (7.50–88.50)	58.00 (11.00–180.00)	51.00 (19.50–91.00)	68.00 (4.50–464.00)	0.619

Data are presented as median (interquartile range, IQR). Overall group differences were assessed using the Kruskal–Wallis test. For variables with significant overall differences, *post-hoc* pairwise Wilcoxon rank-sum tests with Bonferroni correction were performed.

### Video content

**Figure 3:**
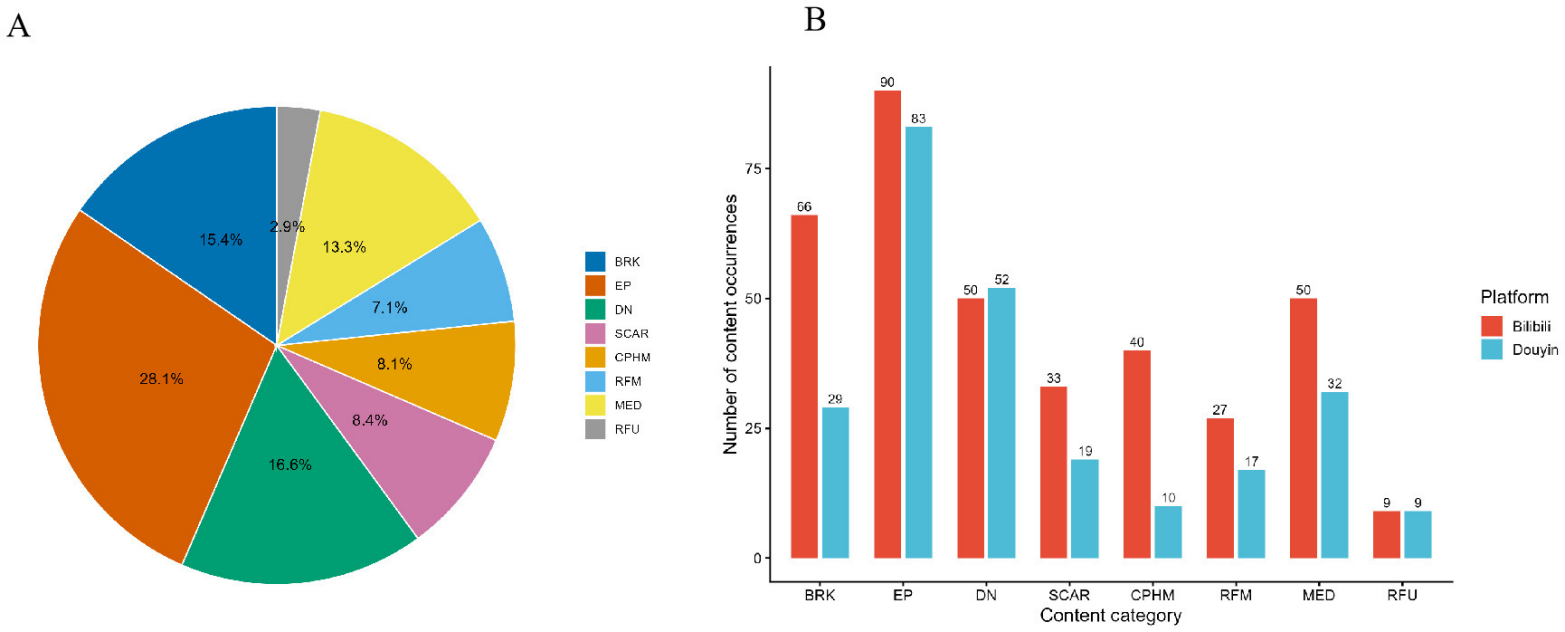
Comparison of content presentation formats. **(A)** Percentage distribution of different content presentation formats. **(B)** Number of different content presentation formats on Douyin and Bilibili. BRK, Basic Rehabilitation Knowledge; EP, Exercise Prescription; DN, Diet and Nutrition; SCAR, Smoking Cessation and Alcohol Reduction; CPHM, Complication Prevention and Health Management; RFM, Risk Factor Control and Monitoring; MED, Medication Management; RFU, Rehabilitation Follow-up.

As shown in Table 3 and Figure 3, the overall distribution of video content occurrences differed significantly between Bilibili and Douyin (χ^2^ = 22.396, *P* = 0.002). Content related to Basic Rehabilitation Knowledge and Complication Prevention and Health Management was proportionally more common on Bilibili than on Douyin (17.98% vs. 11.51%, *P* = 0.02; 10.90% vs. 3.97%, *P* = 0.001). In contrast, Exercise Prescription (32.94% vs. 24.52%, *P* = 0.02) and Diet and Nutrition (21.03% vs. 13.90%, *P* = 0.01) were more frequently represented on Douyin. No statistically significant differences were observed between the two platforms for Smoking Cessation and Alcohol Reduction, Risk Factor Control and Monitoring, or Medication Management and Rehabilitation Follow-up (*P* > 0.05).

**Table 3:** Content characteristics of videos on Bilibili and Douyin.

Platform	Bilibili (*n* = 100)	Douyin (*n* = 100)	*P*
Type of topics, *n* (%)			
Basic rehabilitation knowledge	66 (17.98%)	29 (11.51%)	0.02
Exercise rehabilitation	90 (24.52%)	83 (32.94%)	0.02
Diet and nutrition	51 (13.9%)	53 (21.03%)	0.01
Smoking cessation and alcohol reduction	33 (8.99%)	19 (7.54%)	0.5
Complication prevention and health management	40 (10.9%)	10 (3.97%)	0.001
Risk factor control and monitoring	28 (7.63%)	17 (6.75%)	0.6
Medication management	50 (13.62%)	32 (12.70%)	0.7
Rehabilitation follow-up	9 (2.45%)	9 (3.57%)	0.41
Style of video shooting, *n* (%)			
Solo narration	94 (47.2%)	68 (41.0%)	0.36
Question or answer	3 (1.49%)	7 (4.22%)	0.12
PPT or class	53 (26.5%)	13 (7.83%)	<0.001
Animation or action	27 (13.5%)	32 (19.3%)	0.37
Medical scenarios	5 (2.48%)	37 (22.3%)	<0.001
TV show or documentary	18 (9.0%)	9 (5.42%)	0.28

Data are presented as number (percentage); Type of topics, *n* (%) = (Number of category assignments for a given topic ÷ Total number of category assignments within the platform) × 100. A two-tailed *P*-value <0.05 was considered statistically significant.

**Figure 4:**
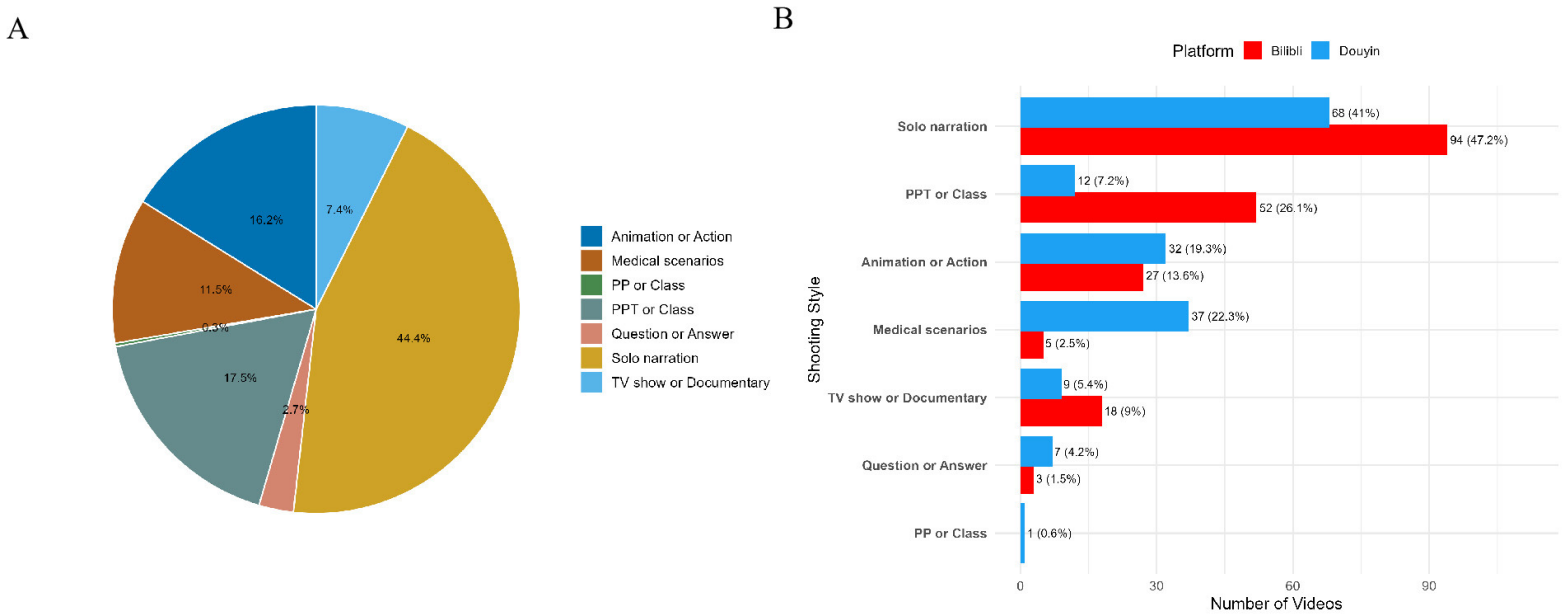
Distribution of video content categories. **(A)** Video presentation styles. **(B)** Comparison of presentation styles between Douyin and Bilibili.

As shown in Table 3 and Figure 4, PPT or Class style videos were significantly more common on Bilibili than on Douyin (26.5% vs. 7.83%, *P* < 0.001), whereas medical scenarios videos were more prevalent on Douyin than on Bilibili (22.3% vs. 2.48%, *P* < 0.001). No significant differences were observed between the two platforms for question or answer, animation or action, and solo narration videos (*P* > 0.05).

### Video quality and reliability assessments

**Figure 5:**
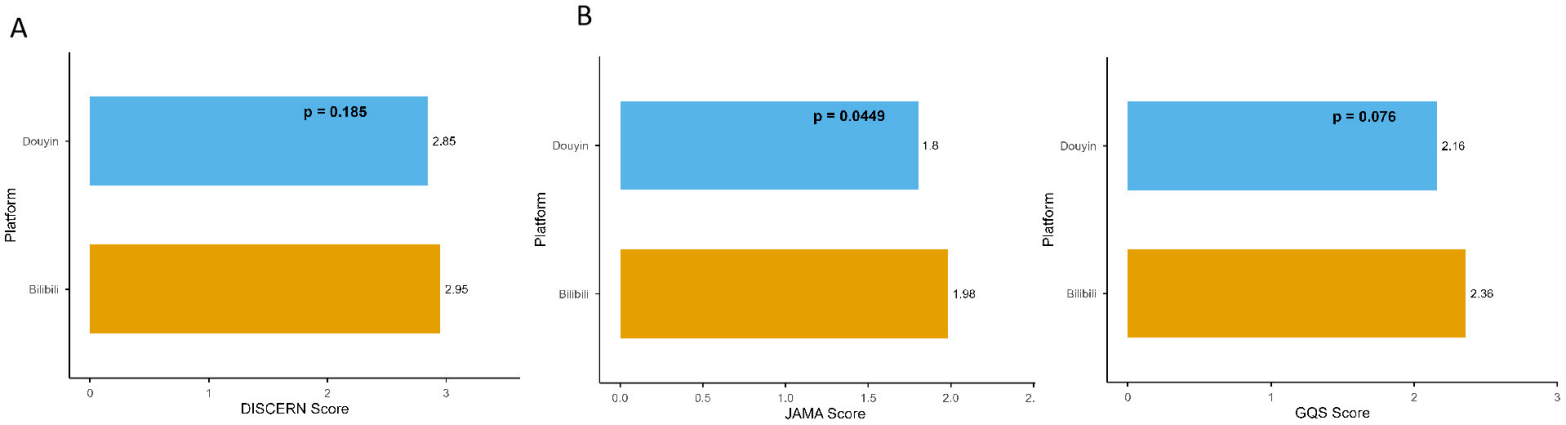
Video quality scores on Bilibili and Douyin. **(A)** Comparison of mDISCERN scores between Bilibili and Douyin; **(B)** Comparison of JAMA scores between Bilibili and Douyin; **(C)** Comparison of GQS scores between Bilibili and Douyin.

Excellent inter-rater reliability was observed across all three scoring systems, supporting the robustness of the quality assessment protocol. The intraclass correlation coefficients (ICCs) were 0.932 (95% CI: 0.908–0.950) for mDISCERN, 0.957 (95% CI: 0.944–0.968) for JAMA, and 0.953 (95% CI: 0.938–0.964) for GQS. At the platform level (Figure 5), Bilibili videos had significantly higher JAMA scores than Douyin videos (*P* = 0.04), whereas no significant differences were observed for mDISCERN (*P* = 0.185) or GQS (*P* = 0.076). These findings suggest that platform-related differences were mainly reflected in information transparency and source attribution rather than overall educational quality.

**Figure 6:**
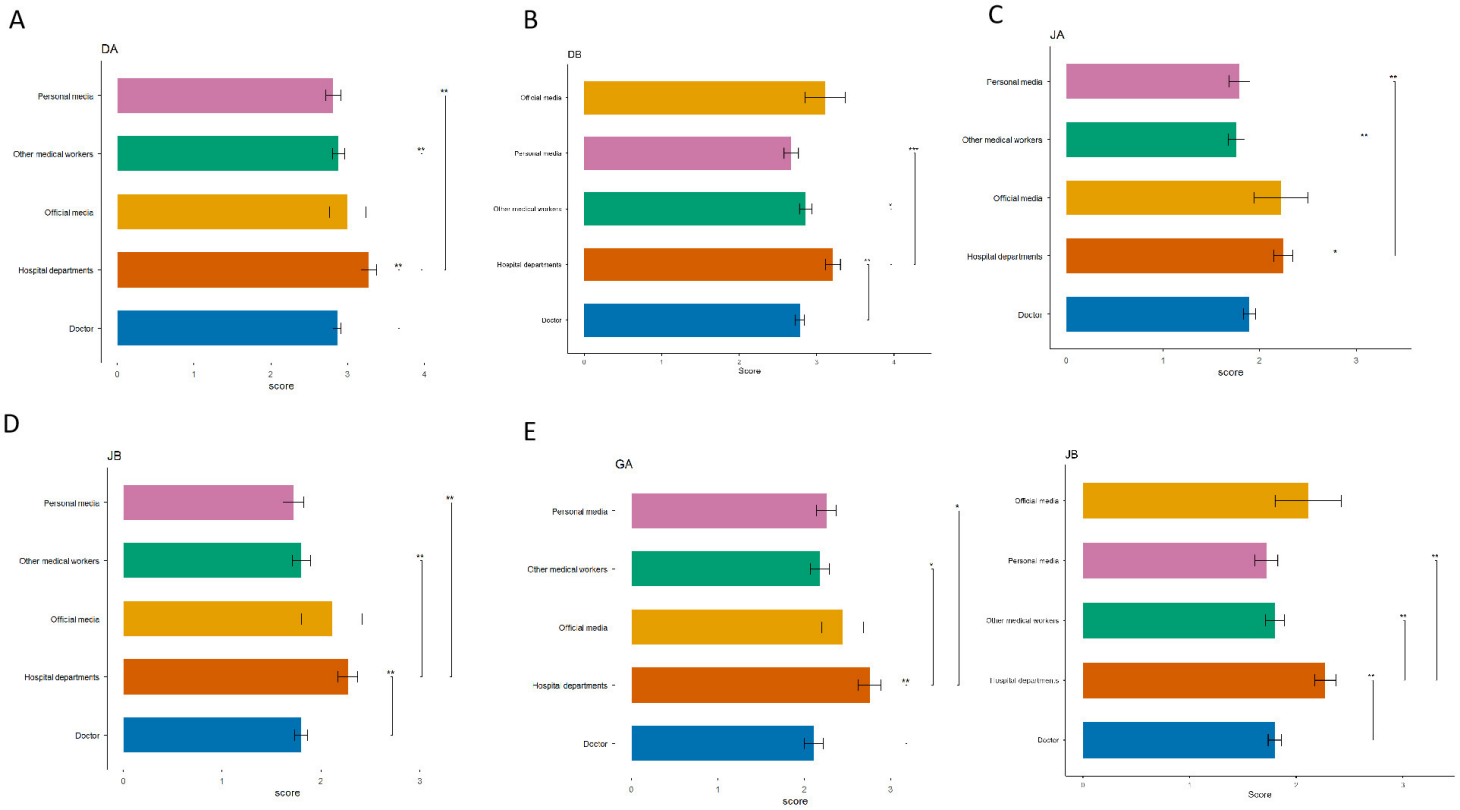
Video source characteristics and quality assessment across uploader categories. **(A, B)** mDISCERN scores assigned by Reviewer A and Reviewer B, respectively; **(C, D)** JAMA scores assigned by Reviewer A and Reviewer B, respectively. **(E, F)** GQS scores assigned by Reviewer A and Reviewer B, respectively. *P < 0.05; **P < 0.01; ***P < 0.001.

**Figure 7:**
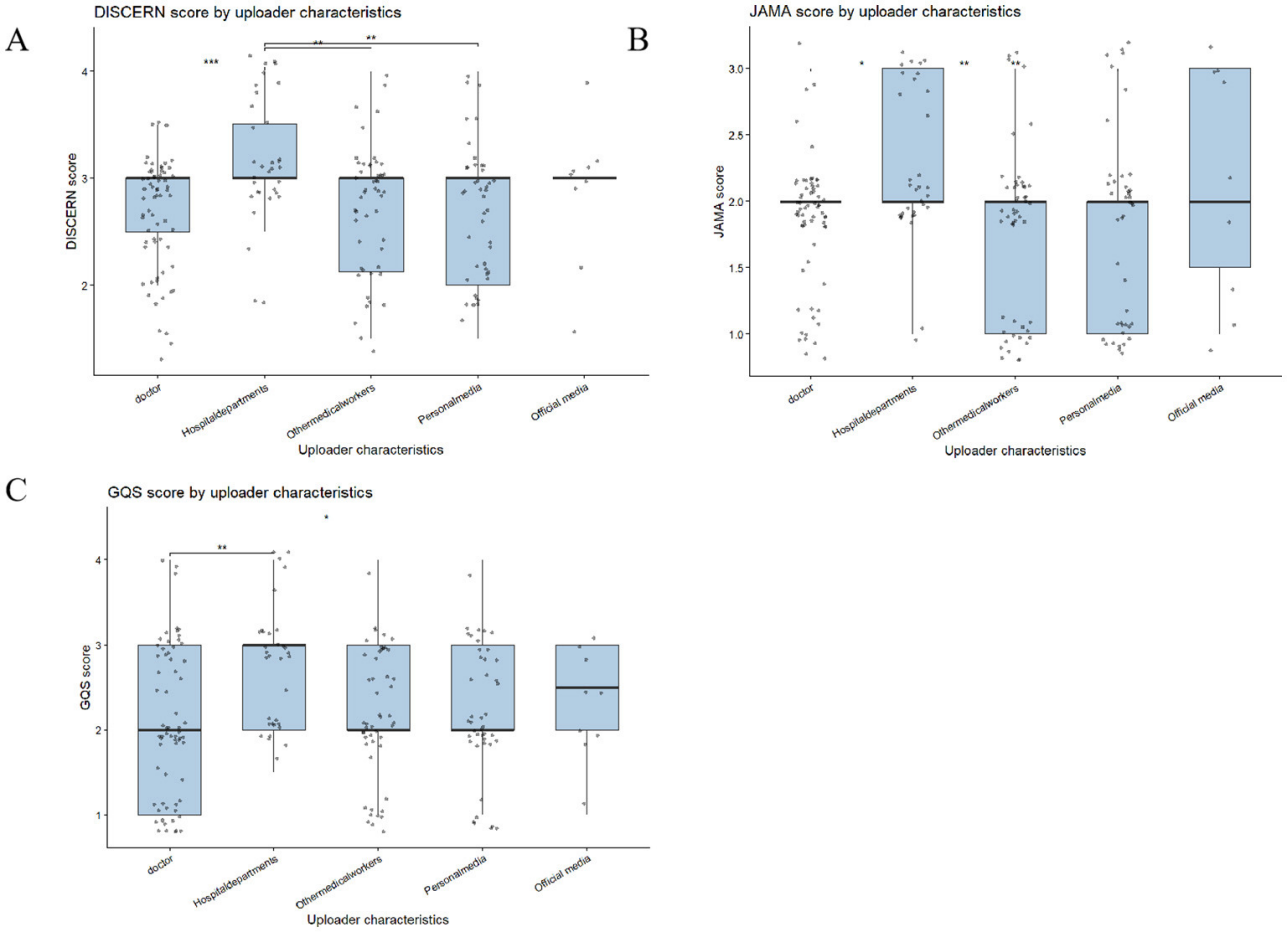
Comparison of video quality scores across different uploader characteristics. **(A)** Videos uploaded by hospital departments showed significantly higher mDISCERN scores compared with personal media (***P < 0.001), doctors (**P < 0.01), and other medical workers (**P < 0.01); **(B)** Hospital departments achieved significantly higher JAMA scores than doctors (*P < 0.05), personal media (**P < 0.01) and other medical workers (**P < 0.01); **(C)** Hospital departments achieved significantly higher GQS scores than doctors (**P < 0.01) and other medical workers (*P < 0.05).

Across uploader categories, When comparing whether there are differences in scores across different uploader types (Figures 6, 7 and Table 4), hospital department accounts consistently achieved the highest video quality scores, with such scores differing significantly across uploader types in all three evaluation systems. Effect size analysis based on η^2^ indicated small to moderate differences among uploader categories (η^2^ = 0.051–0.077), suggesting that although statistically significant, the magnitude of these differences was limited. Both reviewers' median scores (DA and DB) were highest for hospital department videos 3.0 (IQR 3–4). *Post-hoc* analyses confirmed that hospital department videos outperformed those uploaded by doctors, other medical workers, and personal media (*P* < 0.01). Hospital department videos again ranked highest for JAMA 2.0 (IQR 2.0–3.0; *P* = 0.002) and GQS 3.0 (IQR 2.0–3.0; *P* = 0.007). *Post-hoc* comparisons indicated significantly higher JAMA scores compared with other medical workers and personal media, and higher GQS scores compared with doctors and other medical workers.

**Table 4:** Differences in information quality scores (mDISCERN, JAMA, and GQS).

Reviewers' score	Doctor	Other medical workers	Personal media	Hospital department	Official media	*P*	η^2^
DA, median (IQR)	3 (3–3)	3 (3–3)	3 (2–3)	3 (3–4)	3 (3–3)	0.005	0.0550
DB, median (IQR)	3 (3–3)	3 (3–3)	3 (2–3)	3 (3–4)	3 (3–4)	0.0009	0.0744
mDISCERN	3 (2.5–3)	3 (2.125–3)	3 (2–3)	3 (3–3.5)	3 (3–3)	0.0007	0.0767
JA, median (IQR)	2 (2–2)	2 (1–2)	2 (1–2)	2 (2–3)	2 (2–3)	0.002	0.0623
JB, median (IQR)	2 (2–2)	2 (1–2)	2 (1–2)	2 (2–3)	2 (1–3)	0.001	0.0689
JAMA, median (IQR)	2 (2–2)	2 (1–2)	2 (1–2)	2 (2–3)	2 (1.5–3)	0.002	0.0635
GA, median (IQR)	2 (1–3)	2 (2–3)	2 (2–3)	3 (2–3)	3 (2–3)	0.007	0.0511
GB, median (IQR)	2 (1–3)	2 (2–3)	2 (2–3)	3 (2–3)	2 (2–3)	0.007	0.0509
GQS	2 (1–3)	2 (2–3)	2 (2–3)	3 (2–3)	2.5 (2–3)	0.007	0.0515

Data are presented as median (interquartile range, IQR); DA and DB, mDISCERN scores assigned by Reviewer A and Reviewer B, respectively; JA and JB, JAMA scores assigned by Reviewer A and Reviewer B, respectively; GA and GB, GQS scores assigned by Reviewer A and Reviewer B, respectively. *P*-values were calculated using the Kruskal–Wallis test, and effect sizes were expressed as eta-squared (η^2^).

The association between video content quantity and quality was examined. As shown in Table 5, the median number of content components per video was 3 (IQR 2–4). Spearman correlation analysis indicated that content quantity was significantly positively associated with JAMA scores (ρ = 0.168, *P* = 0.018) and GQS scores (ρ = 0.142, *P* = 0.044), while its correlation with mDISCERN scores was not significant (ρ = 0.103, *P* = 0.146). These results suggest that videos with more content may have slightly higher transparency and usability, but overall quality is not determined solely by content quantity.

**Table 5:** Spearman correlations between video content count and quality scores.

Quality score	Median (IQR)	Video content count	Spearman ρ	*P*
mDISCERN	3 (3–3)	3 (2–4)	0.103	0.146
JAMA	2 (1.5–2)	3 (2–4)	0.168	0.018
GQS	2 (2–3)	3 (2–4)	0.142	0.044

Data are presented as Data are presented as median (interquartile range, IRQ). A two-tailed *P*-value <0.05 was considered statistically significant.

**Figure 8:**
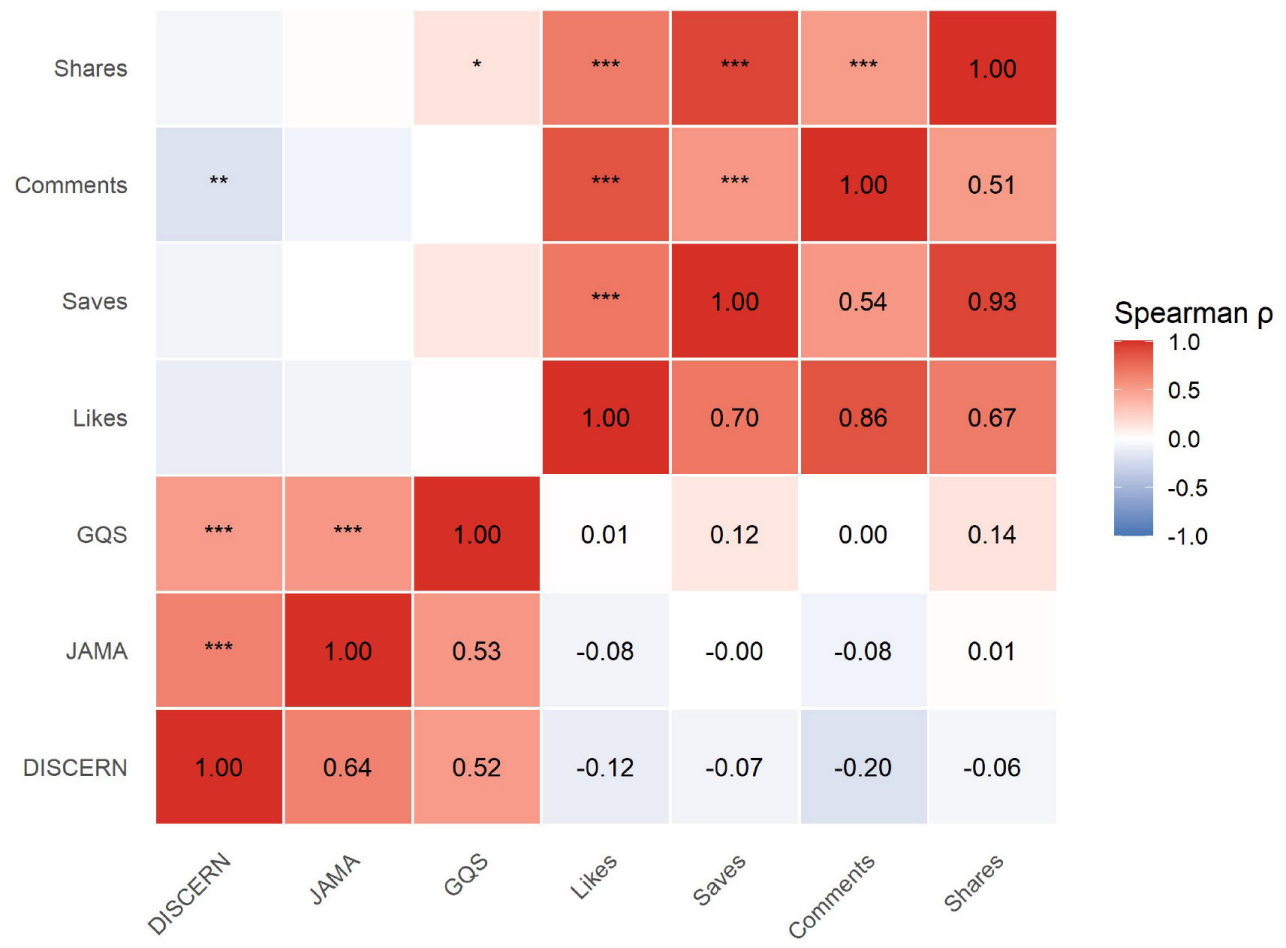
Spearman correlation matrix of cardiac rehabilitation video variables (mDISCERN, JAMA, and GQS) scores. ^*^P < 0.05; ^**^P < 0.01; ^***^P < 0.001.

As shown in Figure 8, video quality scores were weakly and inconsistently associated with user engagement metrics, with only a few reaching statistical significance. Specifically, mDISCERN scores showed a weak negative correlation with the number of comments (ρ = −0.196, *P* = 0.005), while no significant associations were observed with likes, saves, or shares. GQS scores were weakly positively correlated with shares (ρ = 0.141, *P* = 0.047), but showed no significant associations with likes, saves, or comments. In contrast, JAMA scores were not significantly correlated with any engagement metric.

## Discussion

Cardiac rehabilitation (CR) has been widely shown to improve exercise capacity, quality of life, and clinical outcomes in patients with cardiovascular disease, while reducing the risks of rehospitalization and mortality (27–29).

Previous studies have shown that many patients, particularly those after hospital discharge, lack a systematic understanding of the specific components, implementation strategies, and long-term benefits of CR, which in turn limits their active participation and long-term adherence (30, 31). With the rapid development of the internet and social media platforms, the dissemination of medical knowledge and delivery of standardized health education through digital channels such as videos have become important approaches to improving patient awareness, promoting participation in rehabilitation, and enhancing self-management after hospital discharge (32, 33). However, other studies have also pointed out that health-related information on social media is often characterized by insufficient scientific rigor, incomplete content, or non-standard presentation, making it difficult for users to assess the reliability of the information (33). A growing body of research has evaluated the quality of medical information on social media platforms (34, 35). Nevertheless, studies focusing specifically on CR-related video content remain limited. Consistent with previous studies by Tezcan et al. (19) and Karataş et al. (20), the overall quality of CR-related videos was found to be generally low to moderate, suggesting that suboptimal informational quality remains a pervasive issue across video-sharing platforms. The present study, however, extends the existing literature in several meaningful ways. First, the adoption of a cross-platform design provides comparative evidence from both Douyin and Bilibili, offering a broader perspective on the contemporary short-video ecosystem that moves beyond the single-platform focus characteristic of earlier work. Second, beyond overall quality assessment, the study incorporated a detailed analysis of uploader characteristics, content categories, and presentation styles, enabling a more nuanced understanding of the factors shaping information delivery. Third, whereas prior studies have largely described user engagement through basic metrics, the present study systematically examined the associations between engagement indicators and objective quality measures. Crucially, by accounting for differences in video exposure time, a more standardized comparison of dissemination efficiency was achieved—providing new insights into whether higher-quality content is more likely to generate meaningful user interaction, an aspect that has received insufficient attention in the existing literature. Overall, the quality of CR-related content on both platforms was generally suboptimal. Videos on Bilibili achieved significantly higher JAMA scores than those on Douyin, whereas no significant differences were observed in mDISCERN or GQS scores between the two platforms. This finding suggests that, in terms of information transparency, structure, and source attribution, content on Bilibili may adhere more closely to standardized reporting practices. However, perceived usability and overall educational value appeared relatively comparable across the two platforms. Taken together, these results indicate that there are platform-specific differences in the dissemination of CR-related health information through short-video media, while limitations in scientific accuracy, balance, and reliability remain common across platforms. These findings are generally consistent with those reported in previous studies across other medical domains (36, 37).

Uploader characteristics differed markedly between the two platforms. On Douyin, uploaders were predominantly physicians and hospital departments, whereas creators on Bilibili were more diverse. In addition to medical professionals, a substantial proportion consisted of other healthcare workers and personal media or individual content creators, reflecting an audience that is more oriented toward learning-and knowledge-based communities. Further analysis revealed that, despite the higher proportion of professionally affiliated uploaders on Douyin, the information quality of its videos varied considerably. In contrast, videos released by hospital departments performed best across professional evaluation metrics, including mDISCERN, JAMA, and GQS, a finding that is consistent with previous studies (38). These findings suggest that professional institutions may play a more important role in ensuring the standardization, transparency, and reliability of health information. There are observable differences in video quality across uploader types, with hospital department accounts demonstrating more consistent performance and higher overall scores across all evaluation metrics.

This study further revealed clear differences in content focus and presentation formats between the two platforms. Douyin videos placed greater emphasis on action demonstrations and visual appeal, with a higher proportion of content related to exercise rehabilitation and nutrition. In contrast, Bilibili featured a larger share of videos covering fundamental rehabilitation knowledge and complication prevention, primarily presented in PPT-based or classroom-style formats, suggesting a stronger orientation toward structured and systematic health education. These findings are consistent with the conclusions reported by Liang et al. (37). In addition, as the number of content components increased, GQS and JAMA scores showed a modest upward trend, suggesting that videos with richer content may have slightly higher quality; however, the score distributions largely overlapped across groups, indicating that content quantity is not the key determinant of video quality. This suggests that content richness and thematic breadth alone do not necessarily reflect evidence-based depth or medical rigor. These results are in line with previous social media studies on medical topics such as hepatocellular carcinoma, prostate cancer, systemic lupus erythematosus, and radiotherapy (10, 36–38).

From the perspective of user engagement, differences in dissemination patterns were observed between Douyin and Bilibili. Douyin videos received significantly more likes and comments than those on Bilibili, suggesting stronger immediate interaction and emotional engagement on Douyin. No significant differences were observed in saves or shares prior to adjustment, suggesting that retention- and diffusion-oriented behaviors were broadly comparable between the two platforms. However, after normalizing engagement metrics by upload duration—expressed as interaction rates per 30 days—Douyin consistently yielded higher values across all indicators, including likes, comments, saves, and shares. This pattern suggests that the platform differences observed were not attributable to variations in upload timing or content aging, but instead reflect stable, platform-specific differences in user interaction behavior. The correlation between user engagement metrics and information quality scores was consistently weak across both platforms. Notably, mDISCERN scores showed a significant negative correlation with comment volume, while GQS scores demonstrated only a modest positive association with sharing behavior. These findings suggest that high user engagement does not reliably reflect the evidence-based quality of health content. Commenting and sharing behaviors appear to be driven more by content comprehensibility, emotional resonance, and perceived practical relevance than by scientific rigor or the strength of the underlying evidence base. This observation is consistent with a growing body of literature documenting a persistent disconnect between online content popularity and objective information quality (39, 40).

## Strengths and limitations

The strengths of our study are as follows: this study presents the first systematic assessment of popular science videos on CR from two leading short-video platforms in China (Douyin and Bilibili). we retrieved and screened videos based on each platform's default ranking algorithm, ultimately including 200 videos for analysis. This approach ensures that our analysis reflects the content that typical users are most likely to encounter, providing a realistic assessment of video quality and user engagement. The search strategy was designed to achieve broad coverage and followed a rigorous screening process, ensuring that the findings comprehensively reflect the current state of CR dissemination on short-video platforms. For the assessment of video quality, this study employed a multidimensional evaluation approach. All evaluations were conducted independently by two reviewers to enhance the stability and reliability of the results. Moreover, this study extends beyond assessing video quality *per se* to compare differences in content focus and presentation formats across platforms.

The limitations of this study should be acknowledged. First, video retrieval was based on pre-defined keywords and restricted to Douyin and Bilibili at a single time point. Videos using alternative terminology or uploaded after the search date may have been missed, potentially limiting sample completeness and representativeness. Second, although discrepancies between the two reviewers were resolved through discussion and consensus, the classification of video content, uploader type, and presentation style still involved subjective judgment, and formal inter-rater reliability statistics were not calculated. Third, many videos addressed multiple topics simultaneously, and the independent associations between specific content domains, user engagement, and information quality were not explored. In addition, guideline concordance was not systematically assessed, and misinformation or potentially harmful recommendations were not formally evaluated. Future studies should incorporate guideline-based content audits (e.g., ESC, AHA/ACC, AACVPR) and more detailed stratified analyses to provide a more clinically meaningful evaluation. Furthermore, this study primarily adopted a descriptive and comparative design and did not perform multivariable regression analyses to control for potential confounding factors. Finally, video quality assessment relied mainly on expert-based instruments (mDISCERN, JAMA, and GQS), without evaluating audience comprehension or actual information uptake.

## Conclusion

In summary, our analysis of 200 CR-related videos sourced from two major Chinese short-video platforms revealed that the scientific accuracy and informational reliability of the available content remain generally suboptimal. Videos produced by CR physicians or professional organizations demonstrated higher informational value, however, user engagement metrics, including likes, comments, and favorites, did not consistently align with these differences. These findings underscore the limitations of algorithm-driven recommendation systems in promoting high-quality health information. Improving this situation likely requires platforms to bring in more structured expert input and to place greater weight on content quality within their ranking mechanisms, rather than relying mainly on popularity-based signals. At the same time, greater involvement from healthcare professionals and professional bodies in short-video production would help make CR knowledge more accurate, accessible, and ultimately more trustworthy for the public.

## Data Availability

The original contributions presented in the study are included in the article/Supplementary material, further inquiries can be directed to the corresponding author.
