# Supplementary material for: Quality and reliability of cardiac rehabilitation-related short Chinese videos on Douyin and Bilibili: a cross-sectional content analysis
Source: Front Public Health. 2026 Jun 19;14:1797405. doi: 10.3389/fpubh.2026.1797405 (PMC13328417; doi:10.3389/fpubh.2026.1797405)
Supplement: Supplementary file 1 [file Data_Sheet_1.docx]

**Title: Quality and Reliability of Cardiac Rehabilitation–Related Short Chinese Videos on Douyin and Bilibili: A Cross-Sectional Content Analysis**

Supplementary Table 1.Details are provided in Supplementary Table 1

Supplementary Table 2. Characteristics of Videos on Bilibili and Douyin

Supplementary Table 3. Modified DISCERN (mDISCERN) Scoring Criteria

Supplementary Table 4. JAMA Benchmark Criteria for Video Quality

Supplementary Table 5. Global Quality Score (GQS) Criteria

Supplementary Table 6. Dunn’s post-hoc comparisons of video quality scores among uploader types(DISCERN)

Supplementary Table 7. Dunn’s post-hoc comparisons of video quality scores among uploader types(JAMA)

Supplementary Table 8. Dunn’s post-hoc comparisons of video quality scores among uploader types(GQS)

**Table 1.** Characteristics of Videos on Bilibili and Douyin

| Video parameter | Overall  (n = 200) | Douyin  (n = 100) | Huber M-Est | Bilibili  (n = 100) | Huber M-Est | *P* |
| --- | --- | --- | --- | --- | --- | --- |
| **Length, min** | 2.57 (1.28-8.48) | 1.35  (1.00-2.10) | 1.49 | 7.25 (3.00-26.38) | 10.56 | <0.001 |
| **Upload age, days** | **677**  **(207-1303)** | 218.5 (103.25-475.25) | 239.78 | 1268.5 (924-1620.25) | 1257.68 | <0.001 |
| **Likes , n** | 43  (18-205) | 150 (34.25-763.50) | 177.94 | 23  (7.25-47) | 21.25 | <0.001 |
| **Saves, n** | 62  (11-208) | 32  (8.25-220) | 46.68 | 65 (26.25-205.25) | 80.19 | 0.33 |
| **Comments, n** | 3  (1-16) | 8.5  (2.00-39.25) | 11.12 | 1  (0-3) | 1.18 | <0.001 |
| **Shares, n** | 45  (8-141) | 31  (4.25-235) | 39.13 | 54.5 (12.25-106) | 55.58 | <0.001 |

Note: values are presented as median (interquartile range, IQR). Huber M-estimators are reported as robust estimates of central tendency. Group comparisons were performed using non-parametric tests. A two-tailed *P* value < 0.05 was considered statistically significant.

**Supplementary Table2** : Modified DISCERN (mDISCERN) Scoring Criteria

| No | Evaluation Item | Description | Scoring |
| --- | --- | --- | --- |
| 1 | Clarity of aims | Whether the aims of the video are clearly stated and achieved | 1 = Yes, 0 = No |
| 2 | Reliability of sources | Whether the information is supported by reliable, evidence-based sources | 1 = Yes, 0 = No |
| 3 | Balance and bias | Whether the content is presented in a balanced and unbiased manner | 1 = Yes, 0 = No |
| 4 | Additional references | Whether additional references or sources of information are provided | 1 = Yes, 0 = No |
| 5 | Acknowledgement of uncertainty | Whether areas of uncertainty or limitations are acknowledged | 1 = Yes, 0 = No |

**Supplementary Table3:** JAMA Benchmark Criteria for Video Quality

| **Criterion** | **Description** | **Scoring** |
| --- | --- | --- |
| Authorship | Whether the authorship and credentials of the video creator are clearly stated | 1 = Yes, 0 = No |
| Attribution | Whether references or sources of information are provided | 1 = Yes, 0 = No |
| Disclosure | Whether conflicts of interest or funding sources are disclosed | 1 = Yes, 0 = No |
| Currency | Whether the date of upload or last update is provided | 1 = Yes, 0 = No |

**Supplementary Table4.** Global Quality Score (GQS) Criteria

| **Score** | **Description** |
| --- | --- |
| 1 | Poor quality, very little information, limited help for patients |
| 2 | Medium quality, some useful content but overall sub-par |
| 3 | Medium quality, sufficient presentation of key information |
| 4 | High quality, comprehensive content with strong practicality |
| 5 | Excellent quality, extremely informative and highly practical for patients |

**Supplementary Table 5.** Dunn’s post-hoc comparisons of video quality scores among uploader types(DISCERN)

| **Tool** | **Group1** | **Group2** | **n1** | **n2** | **Z statistic** | **Adjusted p** | **Significance** |
| --- | --- | --- | --- | --- | --- | --- | --- |
| DISCERN | Doctor | Hospital departments | 65 | 33 | 3.68 | 0.00231 | ** |
| DISCERN | Doctor | Official media | 65 | 9 | 1.27 | 1.00000 | ns |
| DISCERN | Doctor | Other medical workers | 65 | 50 | 0.49 | 1.00000 | ns |
| DISCERN | Doctor | Personal media | 65 | 43 | -0.61 | 1.00000 | ns |
| DISCERN | Hospital departments | Official media | 33 | 9 | -0.89 | 1.00000 | ns |
| DISCERN | Hospital departments | Other medical workers | 33 | 50 | -3.10 | 0.01960 | * |
| DISCERN | Hospital departments | Personal media | 33 | 43 | -3.92 | 0.00090 | *** |
| DISCERN | Official media | Other medical workers | 9 | 50 | -0.99 | 1.00000 | ns |
| DISCERN | Official media | Personal media | 9 | 43 | -1.56 | 1.00000 | ns |
| DISCERN | Other medical workers | Personal media | 50 | 43 | -1.02 | 1.00000 | ns |

**Supplementary Table 6.** Dunn’s post-hoc comparisons of video quality scores among uploader types(JAMA)

| **Tool** | **Group1** | **Group2** | **n1** | **n2** | **Z statistic** | **Adjusted p** | **Significance** |
| --- | --- | --- | --- | --- | --- | --- | --- |
| JAMA | Doctor | Hospital departments | 65 | 33 | 3.04 | 0.02360 | * |
| JAMA | Doctor | Official media | 65 | 9 | 1.19 | 1.00000 | ns |
| JAMA | Doctor | Other medical workers | 65 | 50 | -0.51 | 1.00000 | ns |
| JAMA | Doctor | Personal media | 65 | 43 | -0.86 | 1.00000 | ns |
| JAMA | Hospital departments | Official media | 33 | 9 | -0.60 | 1.00000 | ns |
| JAMA | Hospital departments | Other medical workers | 33 | 50 | -3.33 | 0.00875 | ** |
| JAMA | Hospital departments | Personal media | 33 | 43 | -3.54 | 0.00400 | ** |
| JAMA | Official media | Other medical workers | 9 | 50 | -1.44 | 1.00000 | ns |
| JAMA | Official media | Personal media | 9 | 43 | -1.62 | 1.00000 | ns |
| JAMA | Other medical workers | Personal media | 50 | 43 | -0.35 | 1.00000 | ns |

**Supplementary Table 7.** Dunn’s post-hoc comparisons of video quality scores among uploader types(GQS)

| **Tool** | **Group1** | **Group2** | **n1** | **n2** | **Z statistic** | **Adjusted p** |
| --- | --- | --- | --- | --- | --- | --- |
| GQS | Doctor | Hospital departments | 65 | 33 | 3.57 | 0.00354** |
| GQS | Doctor | Official media | 65 | 9 | 0.88 | 1.00000ns |
| GQS | Doctor | Other medical workers | 65 | 50 | 0.47 | 1.00000ns |
| GQS | Doctor | Personal media | 65 | 43 | 0.63 | 1.00000ns |
| GQS | Hospital departments | Official media | 33 | 9 | -1.20 | 1.00000ns |
| GQS | Hospital departments | Other medical workers | 33 | 50 | -3.01 | 0.02600* |
| GQS | Hospital departments | Personal media | 33 | 43 | -2.76 | 0.05770ns |
| GQS | Official media | Other medical workers | 9 | 50 | -0.62 | 1.00000ns |
| GQS | Official media | Personal media | 9 | 43 | -0.52 | 1.00000ns |
| GQS | Other medical workers | Personal media | 50 | 43 | 0.18 | 1.00000ns |

*P* values were calculated using the Kruskal-Wallis test to compare scores across different uploader characteristics.
