# Supplementary material for: Quality and reliability of cardiac rehabilitation-related short Chinese videos on Douyin and Bilibili: a cross-sectional content analysis
Source: Front Public Health. 2026 Jun 19;14:1797405. doi: 10.3389/fpubh.2026.1797405 (PMC13328417; doi:10.3389/fpubh.2026.1797405)
Supplement: Supplementary file 2 [file Data_Sheet_2.docx]

Stable1. Post hoc pairwise comparisons of likes across uploader categories (Bonferroni-adjusted Wilcoxon tests)

| **Group1** | **Group2** | **P value** | **Significant** |
| --- | --- | --- | --- |
| Other medical workers | Doctor | 0.0000753 | Yes |
| Personal media | Doctor | 0.0264 | Yes |
| Hospital departments | Doctor | 0.301 | No |
| Official media | Doctor | 0.250 | No |
| Other medical workers | Hospital departments | 0.105 | No |
| Other medical workers | Official media | 1.000 | No |
| Personal media | Hospital departments | 1.000 | No |
| Personal media | Official media | 1.000 | No |
| Personal media | Other medical workers | 1.000 | No |
| Official media | Hospital departments | 1.000 | No |

Stable2. Post hoc pairwise comparisons of comments cross uploader categories (Bonferroni-adjusted Wilcoxon tests)

| **Group 1** | **Group 2** | **p-value** | **Significance** |
| --- | --- | --- | --- |
| Doctor | Hospital departments | 0.082 | ns |
| Doctor | Official media | 1.000 | ns |
| Doctor | Other medical workers | <0.001 | *** |
| Doctor | Personal media | 0.079 | ns |
| Hospital departments | Official media | 1.000 | ns |
| Hospital departments | Other medical workers | 0.870 | ns |
| Hospital departments | Personal media | 1.000 | ns |
| Official media | Other medical workers | 1.000 | ns |
| Official media | Personal media | 1.000 | ns |
| Other medical workers | Personal media | 1.000 | ns |

Stable3. Spearman correlation matrix of cardiac rehabilitation video variables (mDISCERN, JAMA, and GQS) scores

| **Score** | **Engagement** | **Spearman ρ** | **P value** | **Significance** |
| --- | --- | --- | --- | --- |
| DISCERN | Likes | -0.120 | 0.091 | No |
| JAMA | Likes | -0.076 | 0.283 | No |
| GQS | Likes | 0.008 | 0.913 | No |
| DISCERN | Saves | -0.072 | 0.311 | No |
| JAMA | Saves | -0.003 | 0.972 | No |
| GQS | Saves | 0.122 | 0.087 | No |
| DISCERN | Comments | -0.196 | 0.005 | **Yes** |
| JAMA | Comments | -0.082 | 0.246 | No |
| GQS | Comments | 0.002 | 0.977 | No |
| DISCERN | Shares | -0.062 | 0.379 | No |
| JAMA | Shares | 0.013 | 0.859 | No |
| GQS | Shares | 0.141 | 0.047 | **Yes** |
